# The Biologic Response to Polyetheretherketone (PEEK) Wear Particles in Total Joint Replacement: A Systematic Review

**DOI:** 10.1007/s11999-016-4976-z

**Published:** 2016-07-18

**Authors:** Ashley A. Stratton-Powell, Kinga M. Pasko, Claire L. Brockett, Joanne L. Tipper

**Affiliations:** Institute of Medical and Biological Engineering, School of Mechanical Engineering, University of Leeds, Leeds, LS2 9JT UK

## Abstract

**Background:**

Polyetheretherketone (PEEK) and its composites are polymers resistant to fatigue strain, radiologically transparent, and have mechanical properties suitable for a range of orthopaedic applications. In bulk form, PEEK composites are generally accepted as biocompatible. In particulate form, however, the biologic response relevant to joint replacement devices remains unclear. The biologic response to wear particles affects the longevity of total joint arthroplasties. Particles in the phagocytozable size range of 0.1 µm to 10 µm are considered the most biologically reactive, particularly particles with a mean size of < 1 µm. This systematic review aimed to identify the current evidence for the biologic response to PEEK-based wear debris from total joint arthroplasties.

**Questions/purposes:**

(1) What are the quantitative characteristics of PEEK-based wear particles produced by total joint arthroplasties? (2) Do PEEK wear particles cause an adverse biologic response when compared with UHMWPE or a similar negative control biomaterial? (3) Is the biologic response affected by particle characteristics?

**Methods:**

Embase and Ovid Medline databases were searched for studies that quantified PEEK-based particle characteristics and/or investigated the biologic response to PEEK-based particles relevant to total joint arthroplasties. The keyword search included brands of PEEK (eg, MITCH, MOTIS) or variations of PEEK types and nomenclature (eg, PAEK, CFR-PEEK) in combination with types of joint (eg, hip, knee) and synonyms for wear debris or immunologic response (eg, particles, cytotoxicity). Peer-reviewed studies, published in English, investigating total joint arthroplasty devices and cytotoxic effects of PEEK particulates were included. Studies investigating devices without articulating bearings (eg, spinal instrumentation devices) and bulk material or contact cytotoxicity were excluded. Of 129 studies, 15 were selected for analysis and interpretation. No studies were found that isolated and characterized PEEK wear particles from retrieved periprosthetic human tissue samples.

**Results:**

In the four studies that quantified PEEK-based particles produced using hip, knee, and spinal joint replacement simulators, the mean particle size was 0.23 µm to 2.0 µm. The absolute range reported was approximately 0.01 µm to 50 µm. Rod-like carbon particulates and granular-shaped PEEK particles were identified in human tissue by histologic analysis. Ten studies, including six animal models (rat, mouse, and rabbit), three cell line experiments, and two human tissue retreival studies, investigated the biologic response to PEEK-based particles. Qualitative histologic assessments showed immunologic cell infiltration to be similar for PEEK particles when compared with UHMWPE particles in all six of the animal studies identified. However, increased inflammatory cytokine release (such as tumor necrosis factor-α) was identified in only one in vitro study, but without substantial suppression in macrophage viability. Only one study tested the effects of particle size on cytotoxicity and found the largest unfilled PEEK particles (approximately 13 µm) to have a toxic effect; UHMWPE particles in the same size range showed a similar cytotoxic effect.

**Conclusions:**

Wear particles produced by PEEK-based bearings were, in almost all cases, in the phagocytozable size range (0.1–10 µm). The studies that evaluated the biologic response to PEEK-based particles generally found cytotoxicity to be within acceptable limits relative to the UHMWPE control, but inconsistent when inflammatory cytokine release was considered.

**Clinical Relevance:**

To translate new and advanced materials into clinical use more quickly, the clinical relevance and validity of preclinical tests need to be improved. To achieve this for PEEK-based devices, human tissue retrieval studies including subsequent particle isolation and characterization analyses are required. In vitro cell studies using isolated wear particles from tissue or validated joint replacement simulators, instead of manufactured particles, are also required.

## Introduction

Successful clinical performance of total joint arthroplasties (TJAs) can be determined by many factors, including material, biomechanical, and tribologic design considerations. In particular, it has been established that wear and the biologic reactivity of wear particles play a key role in long-term implant survivorship [1]. Wear particles produced by joint arthroplasty materials, in particular ultrahigh-molecular-weight polyethylene (UHMWPE), have been implicated in late aseptic loosening and subsequent joint failure [8, 16, 18, 30, 37, 39]. Immunologic cells such as macrophages phagocytoze the debris material, which initiates the release of inflammatory cytokines and stimulates osteoclastic bone resorption [21]. Particle size, morphology, volume, and composition are associated with biologic reactivity [28]. The specific size and composition of particles most likely to be biologically reactive remain a controversial topic, particularly among similar biomaterials such as UHMWPE, highly crosslinked UHMWPE, and vitamin E highly crosslinked UHWMPE [2, 10, 11, 20, 31, 32, 41, 42]. Particles in the phagocytozable size range of 0.1 µm to 10 µm are considered the most biologically reactive, particularly particles with a mean size of < 1 µm [7, 10, 11, 31, 32, 41]. Once particle size reduces below approximately 50 nm, the biologic response diminishes [28]. A consensus around the role of particle volume and/or dose has not been reached [10, 11, 22, 31, 38, 41, 45].

Polyetheretherketone (PEEK) and its carbon fiber composites were introduced as bearing materials for TJAs in the 1990s [46]. As a result of its resistance to fatigue strain, radiologic transparency, and suitability for common sterilization techniques, unfilled or neat PEEK has already been widely used for spinal instrumentation [25]. Another specific benefit of using PEEK and its composites is its variable stiffness, usually facilitated by carbon fiber supplementation [27]. This principle was demonstrated by the development of carbon fiber-reinforced UHMWPE in the 1970s [40]. Carbon fiber-reinforced UHMWPE performed well tribologically in the laboratory [40] but was less successful in the clinic attributable, in part, to poor fatigue resistance and carbon fiber release [33, 48]. In a similar fashion, the mechanical properties of PEEK can be altered by adding carbon fibers [43]. The elastic modulus of carbon fiber-reinforced PEEK (CFR-PEEK) composites can be tailored to mimic the properties of cortical bone (18 GPa) or titanium alloy (110 GPa). Carbon fiber orientation and length dictate these properties [43]. Perhaps, to mitigate a repeat performance of carbon fiber-reinforced UHMWPE, the implementation of clinically available PEEK-based devices has been slow. Only one carbon fiber-reinforced PEEK (CFR-PEEK) total hip arthroplasty (THA) (ABG II Hip System; Stryker SA, Montreux, CH) [35, 36] and one unfilled PEEK nucleus replacement device (NUBAC™; Pioneer Surgical Technology, Marquette, MI, USA) [5] have been evaluated clinically. However, currently no PEEK-based TJA device has been cleared by the FDA for patient use, although the use of PEEK for cervical disc replacement [49] is under consideration. PEEK composites have shown in vitro wear properties comparable to metal-on-metal bearing couples [5, 38, 46] and are commonly used in trauma implants and spinal fixation devices [25]. Although clinical trials are the gold standard assessment for biologic response, preclinical studies are a vital safeguard for patients and act as a potential predictor of clinical performance. In bulk form, PEEK composites generally are considered to be biocompatible [24, 47]. However, because many TJAs fail as a result of biologic responses to particles, it is imperative to identify whether or not the wear debris produced by PEEK devices is cytotoxic or immunologically reactive.

This systematic review therefore aimed to answer the following questions from preclinical and clinical studies: (1) What are the quantitative characteristics of PEEK-based wear particles produced by TJAs? (2) Do PEEK wear particles cause an adverse biologic response when compared with UHMWPE or a similar negative control biomaterial? (3) Is the biologic response affected by particle characteristics?

## Search Strategy and Criteria

We searched Embase (1947 to October 1, 2015) and Ovid MEDLINE (1946 to Week 1 of October 2015) for the following syntax: (1) (PEEK-OPTIMA or MITCH-PCR or MITCH or MOTIS or NUBAC).ti,ab; (2) (PEEK or PAEK or polyetheretherketone or polyaryletheretherketone or poly ether ether ketone or poly-ether-ether-ketone or poly ether-ether ketone or CFR-PEEK or carbon-fiber reinforced PEEK or carbon-fiber reinforced polyetheretherketone or PEK or carbon nanotube-reinforced PEEK or CNF-PEEK or CNF PEEK or all-PEEK).ti,ab; (3) (hip or knee or spine or spinal or disc or finger or metacarpophalangeal or total joint replacement or arthroplasty or joint replacement$).ti,ab; (4) (particle$ or particulate$ or wear or debris or bulk).ti,ab; (5) (osteoly$ or cytotoxic$ or immunologic response or cytokine$ or macrophage$ or lymphocyte$ or monocyte$ or RANK? or tumor necrosis factor or TNF$ or interleukin or IL$ or $inflammatory).ti,ab; (6) (1) OR (2); (7) (4) OR (5); and (8) (3) AND (6) AND (7).

**Fig. 1:**
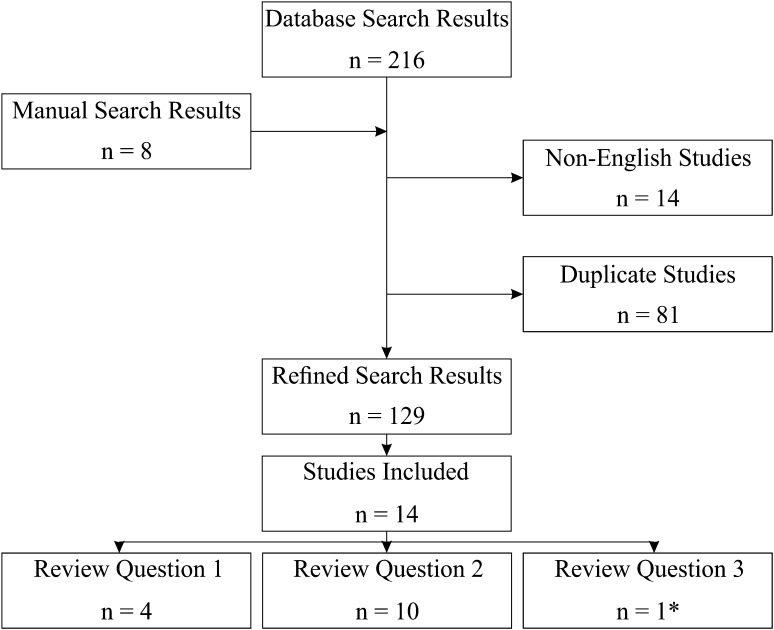
Flow diagram shows the search strategy used. Asterisk denotes studies that were included for more than one question.

The search was limited to peer-reviewed articles published in English. We also searched Google Scholar, reference lists, and conference proceedings using similar terms. Both preclinical and clinical studies (including case studies) were included providing quantitative descriptors of particle characteristics. Articles not relevant to TJAs or reporting biologic responses not relevant to PEEK-based particles (such as contact cytotoxicity studies) were excluded. Two researchers (AAS-P, KMP) reviewed all of the studies independently. A third reviewer (CLB) clarified conflicting decisions (Fig. 1). The initial search retrieved 216 studies, 129 of which were checked, and 14 relevant studies were selected for interpretation and analysis. One study included both a human tissue histology analysis and an animal model.

A range of commercially available PEEK-based materials (Table 1) and custom-made variations were tested across the included studies. There were no detailed characterization studies of particles isolated from retrieved periprosthetic human tissue samples.

**Table 1:** Commercially available PEEK-based materials and products used in the included studies

Material	Commercial name	Manufacturer	Reference
Unfilled PEEK	PEEK-Optima LT1	Invibio Ltd, Thornton-Cleveleys, UK	[6] [13] [14]
CRF-PEEK (Pan)	CFR-PEEK LT1 CA 30	Invibio Ltd	[44] [13] [14]
CRF-PEEK (Pitch)	ABG II Hip System CFR-PEEK LT1 CP 30	Stryker SA, Montreux, CH Invibio Ltd	[35] [26] [44]

CRF-PEEK (Pan) = PEEK containing 30% polyacrylonitrile based carbon fibers; CRF-PEEK (Pitch) = PEEK with a carbon fiber reinforcement of 30% pitch fibers; PEEK = polyetheretherketone.

## Results

Four in vitro studies quantified the characteristics of PEEK-based wear particles produced by TJAs, all of which generated particles in joint replacement simulators [5, 13–15] (Table 2). Four different joint replacements were included. Two types of total disc replacement were tested, one lumbar and one cervical device; and two types of knee replacement were assessed, a unicompartmental knee arthroplasty and a total knee arthroplasty (TKA) designed for patients with metal ion sensitivity. Six different bearing couples were analyzed, two of which featured in more than one study. Feret’s diameter, the distance between two parallel planes constraining each particle, was the most commonly reported descriptor of particle size. The mean particle size (Feret’s diameter) reported by the studies was 0.23 µm to 2.0 µm with the absolute range of approximately 0.01 µm to 50 µm. Self-mating unfilled PEEK bearings were tested for both cervical and lumbar disc replacement devices. Lumbar disc replacement devices produced larger particles than the cervical disc devices using the same bearing materials. CFR-PEEK (Pan)-on-cobalt chromium alloy bearings were tested in both knee arthroplasty studies. The size and morphology of particles were similar but the origin of particles was different. For example, Grupp et al. [15] measured particles from the primary articulation, whereas Grupp et al. [13] analyzed debris from bushings and flanges used within the TKA design.

**Table 2:** Quantitative characteristics of PEEK-based particles produced using clinically relevant joint replacement simulators

Study	Joint	Bearing couples	Feret’s diameter (µm)	ECD (µm)	Range (µm)	AR	E	R	FF
Brown et al. [5]	L.Disc	PEEK/PEEK (UD) PEEK/PEEK (MD)	0.87 2.00	0.69 1.09	–	1.36 1.42	–	0.79 0.75	0.89 0.88
Grupp et al. [12]	C.Disc	UHMWPE/CoCr PEEK/PEEK CRF-PEEK (Pan)/CRF-PEEK (Pan) PEK/PEK	0.13–0.24 0.23–0.51 0.27–0.35 1.0–1.5	–	0.01 to 1-15 0.01 to 1-15 0.01 to 1-15 0.2–50	–	–	–	–
Grupp et al. [15]	UKR	UHMWPE/CoCr CRF-PEEK (Pitch)/CoCr CRF-PEEK (Pan)/CoCr	0.72 ± 0.99 1.27 ± 5.18 0.98 ± 1.75	–	–	1.77 ± 0.94 1.69 ± 0.81 1.65 ± 0.65	3.89 ± 2.88 3.46 ± 2.21 3.12 ± 1.61	0.54 ± 0.21 0.58 ± 0.22 0.61 ± 0.24	0.55 ± 0.14 0.57 ± 0.12 0.59 ± 0.11
Grupp et al. [13]	TKA^*^	CFR-PEEK (Pan)/ZrN CFR-PEEK (Pan)/CoCr	1.89 ± 2.54 0.82 ± 1.80	–	0.05 to 3-20 0.05 to 3–20	1.47 ± 0.48 1.70 ± 0.72	2.23 ± 1.60 3.29 ± 2.52	0.64 ± 0.17 0.58 ± 0.22	–

^*^Only the bushings and flanges within this device were manufactured out of CFR-PEEK; each column represents a mean value; PEEK = polyetheretherketone; L.Disc = lumbar disc; C = disc, cervical disc; UKA = unicompartmental knee arthroplasty; UD = unidirectional motion; MD = multidirectional motion; CoCr = cobalt chromium alloy; ZrN = zirconium nitride; ECD = equivalent circle diameter; AR = aspect ratio; E = elongation ratio; R = roundness; FF = form factor.

Ten studies investigated the biologic response to PEEK-based particles [6, 12, 17, 19, 23, 26, 29, 34, 35, 44]. Two studies performed histologic analysis on retrieved human tissue from failed THAs [26, 35] (Table 3). Evidence of rod-like and granular particles phagocytozed by macrophages was reported but not attributed to wear debris-induced failure. Six studies used in vivo animal models (rat, mouse, and rabbit) to investigate responses to PEEK-based particles [6, 12, 23, 26, 29, 44] (Table 4). All three studies focusing on the spine identified a mild inflammatory response that was local to the particles within the epidural space. Using qualitative histologic descriptions, the immunologic response was not different from the UHMWPE particle groups with the number of studies available [6, 12, 23]. Cunningham et al. [6] was the only animal study to show a reduction in expression of inflammatory cytokines associated with unfilled PEEK particles when compared with UHMWPE particles of the same size. Two studies analyzed particles composed of two CFR-PEEK composites, CFR-PEEK (Pitch) and CFR-PEEK (Pan). (Pitch-based carbon fibers are produced using coal tar pitch and pan-based carbon fibers use polyacrylonitrile [Pan] as an initial processing material [9]. Pitch-based and Pan-based composites exhibit different mechanical properties and can be tailored by the manufacturer [43].) Depending on the cytokine (interleukin [IL]-1β, IL-6, or tumor necrosis factor-α) and area investigated (bone marrow, synovium, or cartilage), the increase of cytokine release cause by CFR-PEEK (Pitch) ranged between two- and sevenfold when compared with the UHMWPE control [29]. To a lesser and statistically significant extent, CFR-PEEK (Pan) particles caused an increase in cytokine expression in bone marrow tissue relative to UHMWPE particles. This was the only study to report adverse tissue reactions to PEEK-based particles when compared with UHMWPE particles. Three studies used in vitro cell lines to assess the biocompatibility of PEEK-based particles [17, 19, 34] (Table 5). Two studies found that PEEK-based particles performed similarly to those of UHMWPE particles, ie, did not display evidence of cytotoxicity elicited by the particles [19, 34]. Hallab et al. [17] reported a reduction in both lactate dehydrogenase (LDH) activity and inflammatory cytokine release for PEEK particles relative to UHMWPE particles, which suggested PEEK particles were less biologically reactive than UHMWPE particles in this study.

**Table 3:** Human tissue retrieval studies after total joint arthroplasty failure

Study	Joint	Device	Bearing couple	Tissue type	Analysis	Outcomes
Latif et al. [26]^*^	THA	ABG II, Stryker Orthopaedics, Mahwah, NJ, USA	CFR-PEEK/alumina	Synovium	Histology	Implantation time: not specified Outcomes: visual inspection; histology (stain not specified) Results: tissue was gray/black but no synovial hypertrophy; histology showed connective tissue of varying density with dark material present within; phagocytozed particles evident within macrophages; rod-like particles and smaller granular particles present; no accumulation of lymphocytes or leucocytes around blood vessels
Pace et al. [35]^*^	THA	ABG II, Stryker Orthopaedics	CFR-PEEK/alumina	Granulomatous periprosthetic tissue	Histology	Implantation time: 26 months Outcomes: histology (stain not specified) Results: connective tissue of varying density evident with areas of highly vascularized granulation tissue; neutrophilic granulocyte, lymphocyte, and plasma cell infiltration evident; perivascular macrophages contained small highly reflective metal particles, larger black particles suggested to be carbon and colorless granular particles exhibiting intense birefringence thought to be of polymer origin

* Retrieved tissue from participants of the same clinical trial; CFR-PEEK = carbon fiber-reinforced polyetheretherketone.

**Table 4:** In vivo animal model studies testing biocompatibility

Study	Animal	Model	Test material	UHMWPE comparator?	Outcomes	Relative reactivity
Latif et al. [26]	Rat	Air pouch	CFR-PEEK	Yes^*^	Test intervals: 1, 3, and 10 days; outcomes: visual scoring system; pouch thickness; localization of macrophages (ED1 antigen staining); vascular proliferation (ICAM1 staining); results: visual scoring system showed PE to cause more inflamed than CFR-PEEK; no differences in pouch thickness for all variables; no difference between polyethylene and CRF-PEEK for ED1 staining or ICAM1 staining	–
Kabir et al. [23]	Rabbit	Epidural	PEK	Yes	Test intervals: 3 and 6 months; outcomes: neurobehavioral observations (weekly); inflammation identified via histology; results: no neurological deficits or systemic toxicity; crystalline wear debris identified and surrounded by inflammatory cells; inflammation and angiogenesis limited to periparticle epidural space	–
Utzschneider et al. [44]^†^	Mouse	Knee	CRF-PEEK (pitch) CRF-PEEK (Pan)	Yes	Test intervals: 1 week; outcomes: synovial microcirculation assessment; fraction of rolling leukocytes; histology (H&E); synovial membrane thickness; results: no difference in functional capillary density, fraction of rolling leukocytes, nor the number of leukocytes adhered to the endothelium between particle groups; no differences in histological scoring for inflammation or synovial membrane thickness were identified between particle groups; the control group had a significant reduction in each of the aforementioned variables compared with the particle groups	–
Cunningham et al. [6]	Rabbit	Epidural	PEEK	Yes	Test intervals: 3 and 6 months; outcomes: histology (H&E and HAM-56 staining); cytokine analysis (ABC method; TNF-α, TNF-β, IL-1α, IL-1β, and IL-6); results: PEEK exhibited a reduced cytokine expression relative to UHMWPE	(−)
Lorber et al. [29]^†^	Mouse	Knee	CRF-PEEK (pitch) CRF-PEEK (Pan)	Yes	Test intervals: 1 week; outcomes: cytokine analysis (ABC method; TNF-α, IL-1β, and IL-6); results: CFR-PEEK (pitch) particles showed significantly increased expression of: TNF-α, IL-1β, and IL-6 in articular cartilage and bone marrow, and TNF-α in the synovial layer when compared with the UHMWPE group; CFR-PEEK (Pan) particles caused increased TNF-α and IL-1β levels in bone marrow compared with UHMWPE particles	(+)
Grupp et al. [12]	Rabbit	Epidural	CRF-PEEK	Yes	Test intervals: 3 and 6 months; outcomes: histology (stain not specified); results: wear debris particles surrounded by inflammatory cells were identified in the vertebral canal; inflammation was limited to the epidural space; CFR-PEEK showed a similar histopathological reaction to UHMWPE particles	–

* Type of polyethylene not specified; ^†^related studies; (+) = increased reactivity; (−) = decreased reactivity; – = similar reactivity; reactivity was judged relative to the within study UHMWPE control; CFR-PEEK = carbon fiber-reinforced polyetheretherketone; ICAM1 = intercellular adhesion molecule 1; H&E = hematoxylin and eosin; TNF = tumor necrosis factor; IL = interleukin.

**Table 5:** In vitro cell line biocompatibility studies

Study	Test material	Particle production	Assay	UHMWPE comparator?	Cell line	Outcomes	Relative reactivity
Morrison et al. [34]	PEEK	Chemical extraction	LDH activity GSH content MTT viability	Yes^*^	3T3 Mouse fibroblasts Rat osteoblasts	Osteoblast morphology remained unchanged after particle exposure for 48 hours; PEEK particles had no significant effect on GSH levels in fibroblasts, a slight decrease in osteoblast GSH levels, and no significant change to LDH activity in either cell line; PEEK exhibited a slight stimulatory effect on osteoblast protein content	–
Howling et al. [19]	CFR-PEEK	Multidirectional Pin-on-plate	ATP viability	No (latex)	L929 Mouse fibroblasts U937 Macrophage-like cells	No cell viability reduction caused by CFR-PEEK particles	–
Hallab et al. [17]	X-UHMWPE PEEK	Cryomilling/pulverization	Proliferation assay ATP viability LDH activity Cytokine analysis	Yes	Human (THP-1) macrophages/monocytes PBMNCs CD14+ monocytes	No significant effects on proliferation or viability between particle types; UHMWPE particles caused greater LDH activity compared with PEEK and X-UHMWPE. X-UHMWPE caused a significant increase in inflammatory cytokines relative to PEEK; the largest PEEK particles (approximately 13 μm) resulted in significant reduction of macrophage viability	(−)

* PE type not specified; PEEK = polyetheretherketone; PE = polyethylene; X-UHMWPE = crosslinked UHMWPE; ATP = adenosine triphosphate; MTT = MTT (3-[4,5-dimethylthiazol-2-yl]-2,5-diphenyltetrazolium bromide); PBMNC = peripheral blood mononuclear cells; LDH = lactate dehydrogenase; GSH = glutathione; (−) = decreased reactivity relative to comparator; – = similar reactivity to comparator.

The in vitro study by Hallab et al. [17] was the only study to associate particle characteristics with biologic response. Unfilled PEEK particles, in the largest size range (approximately 13 µm), reduced macrophage viability, but by no more than 20% of the “medium only” control group (ie, cells with no particles). In the same study, UHMWPE particles, regardless of size, also caused cytotoxic effects. No study directly compared unfilled PEEK against a CFR-PEEK composite, which may reflect manufacturer application preferences for each PEEK type. All of the laboratory studies included in this review used commercially purchased or processed particles that were produced predominantly by cryomilling and/or cryopulverization. Only five of the studies reported quantitative particle characteristics for their biocompatibility testing, four of which used particles produced by cryomilling or cryopulverization [6, 12, 17, 29, 44] (Table 6). Grupp et al. [12] and Utzschneider et al. [44] artificially manufactured particles to replicate their simulator-generated particles for both size and morphology.

**Table 6:** Quantitative characteristics of PEEK-based particles used in the biocompatibility studies

Study	Particle production	Material	Feret’s diameter (µm)	ECD (µm)	Range (µm)	AR	E	R	FF
Utzschneider et al. [44]^*^	Cryopulverization	UHMWPE CRF-PEEK (pitch) CRF-PEEK (Pan)	0.79 ± 0.3 1.22 ± 0.5 1.39 ± 0.3	–	–	1.65 1.61 1.60	–	0.51 0.51 0.54	0.63 0.63 0.66
Hallab et al. [17]^†^	Cryomilling/pulverization	UHMWPE X-UHMWPE PEEK	0.5 ± 0.2; 1.7 ± 0.8; 13 ± 5 0.3 ± 0.2; 2.2 ± 0.9; 10 ± 4 0.7 ± 0.3; 2.4 ± 1.1; 13 ± 6	–	–	1.1–4 1.1–4 1.1–4	–	–	–
Cunningham et al. [6]	Cryomilling/pulverization	UHMWPE PEEK	1.2 0.8	–	0.6–11.0 0.5–18.5	–	–	–	–
Lorber et al. [29]^*^	Cryopulverization	UHMWPE CRF-PEEK (pitch) CRF-PEEK (Pan)	0.79 ± 0.3 1.22 ± 0.5 1.39 ± 0.3	–	–	1.65 1.61 1.60	–	0.51 0.51 0.54	0.63 0.63 0.66
Grupp et al. [12]^‡^	Not specified	UHMWPE CRF-PEEK (Pan)	0.13 to 0.24 0.27 to 0.35	–	0.01 to 1-15 0.01 to 1-15	–	–	–	–

* Particles originated from the same study; ^†^study tested three size ranges per material; ^‡^particles originated from Grupp et al. [15]; each column represents a mean value; PEEK = polyetheretherketone; X-UHMWPE = crosslinked UHMWPE; ECD = equivalent circle diameter; AR = aspect ratio; E = elongation ratio; R = roundness; FF = form factor.

## Discussion

Human tissue particle isolation studies are required to definitively determine TJA wear particle characteristics. No such study was identified and therefore in vivo particle characteristics have not been confirmed or included in this review. However, validated TJA wear simulation is an accepted methodology used in the generation of wear debris associated with specific TJAs. This review identified that there is a lack of clinical studies focused on the wear particles produced by PEEK-based TJAs; therefore, wear simulation and preclinical studies formed the majority of studies used to answer the three main questions: (1) What are the quantitative characteristics of PEEK-based wear particles produced by TJAs? (2) Do PEEK wear particles cause an adverse biologic response when compared with UHMWPE or a similar negative control biomaterial? (3) Is the biologic response affected by particle characteristics? From the relatively small number of studies included, it was found that wear debris produced by PEEK-based bearings was within the phagocytozable size range (0.1–10 µm) and exhibited comparable cytotoxic effects to UHMWPE particles despite a varied cytokine response across the studies.

Several limitations were apparent as we surveyed the available evidence. No studies to date have isolated and characterized particles from retrieved human periprosthetic tissue. Therefore, the true clinical relevance of particle size and morphology distributions produced by simulators for each joint remains unknown. Although Utzschneider et al. [44] and Grupp et al. [12] replicated simulator-generated particle size distributions and morphology accurately, particle production methods such as cryomilling or cryopulverization may not produce relevant particle surface characteristics that are important for particle cell membrane interactions for biologic response experiments. The negative control in most studies consisted of UHMWPE particles. PEEK is an alternative bearing material to more modern UHMWPE formulations (such as highly crosslinked UHMWPE and vitamin E infused or other antioxidant-containing highly crosslinked UHMWPE) and hard-on-hard bearings. Therefore, using conventional UHMWPE particles as a control does not compare like for like with the other commonly used alternatives to PEEK. The biologic response identified in human histology studies such as the investigation by Pace et al. [35] was confounded by particles produced from other interfaces of the joint replacement device, eg, the fixation surface. Emulation of in vivo conditions with in vitro experiments is a challenge. Each study assessing cytotoxicity used different cell lines and varying particle doses and volumes making comparisons between studies inappropriate. Moreover, current polymer isolation methods exploit material density to separate wear debris particles from proteins (ie, UHMWPE particles are buoyant in water, whereas proteins sink). Human proteins and PEEK particles have similar densities (approximately 1.3 g/cc), meaning a new particle isolation method may be required to retrieve PEEK particles from tissue samples or simulator lubricant.

The mean particle size for PEEK-based material bearing couples was within the 0.1-µm to 10-µm size range limit, which is generally accepted as the most biologically reactive. The absolute range reported was approximately 0.01 µm to 50 µm. Most devices produced particles in the submicron size range, which was consistent with other polymer articulations such as UHMWPE-on-cobalt chromium alloy [14, 15]. However, these particle characteristics were determined from only four simulator studies and from devices that are not commonly implanted. The two total disc replacements, one lumbar device and one cervical device, both self-mating unfilled PEEK implants, showed up to a 8.7 times difference in mean particle diameter. The lumbar total disc replacement had the largest mean particle size of the two disc replacement types (2 µm), possibly as a result of different testing protocols (such as using higher loads and greater ROM) relative to the cervical total disc replacement [3]. CFR-PEEK (Pan)-on-cobalt chromium alloy was another bearing couple investigated, although one joint was a unicompartmental knee with a CFR-PEEK (Pan) primary articulation. The other joint was a TKA developed for patients with metal ion sensitivity and used CFR-PEEK (Pan) for bushings and flanges. The wear particles produced by the two devices were similar in size and morphology despite the method of articulation being substantially different. The similarity in particle characteristics may indicate a common wear mode, although a comprehensive damage mode assessment would be required to draw such conclusions and is a recommendation for future studies. Unlike conventional THA and TKA simulation, the clinical relevance of the particles produced in simulator studies analyzing experimental joint replacement devices and/or using novel biomaterials such as PEEK needs to be validated against wear debris isolated from retrieved human tissue samples. No human tissue PEEK-based wear particle isolation studies were identified within this review.

In vivo histopathologic analyses were the most prevalent biocompatibility testing mode used for PEEK-based particles. Histology is a useful tool to retrospectively identify the end condition of the tissue sample location. The two human tissue sample retrieval studies were case studies, each with a sample size of one. Although neither study reported an immunologic response as the cause of failure, generalizable conclusions based on these case studies cannot be made. The animal studies included in this review showed PEEK particles have a minimal effect on immunologic cell recruitment, an indicator of immunologic response, when compared with UHMWPE particles. A similar immunologic response, identified using histology, was reported for unfilled PEEK wear particles produced by spinal instrumentation [39]. Despite the majority of the animal studies showing a comparable biologic response between particle groups, the range of different methods and anatomic test locations means the results cannot be generalized with the limited literature available. The in vitro cytotoxicity studies reported either no or low suppression of macrophage viability and no substantial changes to LDH activity or glutathione content. Different assays and cell lines were used between studies, which limited relevant comparisons. The clinical relevance of artificially manufactured particles used in animal studies requires further investigation.

Particle size, morphology, volume (or dose), and composition are associated with biologic reactivity [29]. Hallab et al. [17] was the only study included in this review to test particle size effects on cytotoxicity. It is generally accepted that particles in the phagocytozable size range (0.1–10 µm) are the most biologically reactive [7, 10, 11, 31, 32, 41]; however, Hallab et al. [17] found the largest PEEK particles (approximately 13 µm) to have a cytotoxic effect. However, in the same study, UHMWPE particles, regardless of size, also caused cytotoxic effects. These results should be viewed with caution, because UHMWPE should not have adverse effects on cell viability. This may be a consequence of the particle manufacturing process. For instance, cryomilling often uses a surfactant during the milling process that can be degraded (eg, serum) or toxic to cells (eg, oleic acid). The cytotoxicity observed for both the UHMWPE and PEEK particles is therefore likely a consequence of contamination. The clinical relevance of PEEK particles in the > 10-µm size range is yet to be determined, although at least two of the simulator studies identified by this review isolated and characterized particles in the > 10-µm size range. Other factors to consider when interpreting particle biocompatibility studies include whether the particles produced by cryomilling/pulverization differ in surface characteristics (eg, surface topography and the presence of endotoxins) that could have an effect on cell behavior. Additionally, a complex relationship exists among particle size, debris volume, and tissue volume that has not been completely elucidated for UHMWPE particles [10, 11, 22, 31, 38, 41, 45] let alone PEEK particles, which may provide additional factors to consider independently of particle size and morphology such as surface chemistry (eg, hydrophilicity) or the prescence of carbon fiber particles and their characteristics.

Polyetheretherketone and its carbon composites are polymers that have customizable mechanical properties suitable for orthopaedic applications [43], high-performing in vitro biotribologic properties [4], and are biocompatible in bulk form [24, 47]. However, in light of CFR-UHMWPE device failure in the decades before the implementation of the current preclinical testing standards [48], the biologic response to PEEK wear debris must be investigated. From the published evidence included in this review [5, 6, 12–15, 17, 19, 23, 26, 29, 34, 35, 44], wear particles produced by PEEK-based bearings in TJA wear simulators were, in almost all cases, in the phagocytozable size range (0.1–10 µm). Despite this, the biologic response to PEEK-based particles has thus far been generally found not to cause cytotoxic effects, but was variable when considering inflammatory cytokine release. It should be noted that only 10 studies were identified in this review to have investigated the biologic response to PEEK-based particles from TJAs, all of which used model particles or particles generated by cryomilling/cryopulverization. Before preclinical assessments of biologic response can accurately reflect the cell interactions with PEEK-based particles, in vivo generated wear particle characteristics must be determined. The first step to identifying clinically relevant particle characteristics is to perform human tissue retrieval studies with subsequent particle isolation and characterization analyses. None have been completed for PEEK-based TJA wear particles thus far. Once the wear particle characteristics have been identified, the size and morphologies can be emulated by joint replacement simulators, isolated, and immunologically tested in cell studies. These steps are required before the biologic response to PEEK-based particles can be determined accurately and repeatably.
